# Constructing TC-1-GLUC-LMP2 Model Tumor Cells to Evaluate the Anti-Tumor Effects of LMP2-Related Vaccines

**DOI:** 10.3390/v10040145

**Published:** 2018-03-23

**Authors:** Liying Sun, Yanzhe Hao, Zhan Wang, Yi Zeng

**Affiliations:** State Key Laboratory for Infectious Disease Prevention and Control, Chinese Center for Disease Control and Prevention, National Institute for Viral Disease Control and Prevention, Beijing 100052, China; sunliying@emails.bjut.edu.cn (L.S.); haoyanzhe@ivdc.chinacdc.cn (Y.H.)

**Keywords:** Epstein-Barr virus, latent membrane protein 2, *Gaussia luciferase*, cytotoxic T lymphocyte, in vivo imaging system

## Abstract

Epstein-Barr virus (EBV) is related to a variety of malignant tumors, and its encoded protein, latent membrane protein 2 (LMP2), is an effective target antigen that is widely used to construct vector vaccines. However, the model cells carrying LMP2 have still not been established to assess the oncolytic effect of LMP2-related vaccines at present. In this study, TC-1-GLUC-LMP2 tumor cells were constructed as target cells to evaluate the anti-tumor effects of LMP2-assosiated vaccines. The results showed that both *LMP2* and *Gaussia luciferase* (*GLuc*) genes could be detected by polymerase chain reaction (PCR) and reverse transcription-polymerase chain reaction (RT-PCR) in TC-1-GLUC-LMP2 cells. Western blot results showed that the LMP2 and *Gaussia luciferase* proteins were stably expressed in tumor cells for at least 30 generations. We mixed 5 × 10^4^ LMP2-specific mouse splenic lymphocytes with 5 × 10^3^ TC-1-GLUC-LMP2 target cells and found that the target cells were killed as the specific killing effect was obviously enhanced by the increased quantities of LMP2-peptide stimulated spleens. Furthermore, the tumor cells could not be observed in the mice inoculated TC-1-GLUC-LMP2 cells after being immunized with vaccine-LMP2, while the vaccine-NULL immunized mice showed that tumor volume gradually grew with increased inoculation time. These results indicated that the TC-1-GLUC-LMP2 cells stably expressing *LMP2* and *GLuc* produced tumors in mice, and that the LMP2-specific cytotoxic T lymphocyte (CTL) effectively killed the cells in vitro and in vivo, suggesting that TC-1-GLUC-LMP2 cells can be used as model cells to assess the immune and antitumor effects of LMP2-related vaccines.

## 1. Introduction

Epstein-Barr virus (EBV) was the first human carcinogenic virus to be discovered, and it affects more than 5 billion people worldwide; up to 200,000 new cases of EBV-related malignancies are diagnosed each year. Therefore, EBV prevention and the treatment of associated cancers have become worldwide public health issues [1,2]. Most EBV patients are related to latency infections, and tumor cells typically expressed nine viral proteins, including six nuclear antigens (EB nuclear antigens, (EBNAs) 1, 2, 3a, 3b, 3c, and -leader protein (-LP)), three latent membrane proteins (LMPs) 1, 2a and 2b, as well as EBV-encoded RNAs and BamHI-A rightward transcripts (BARTs) [3,4]. Specific EBV latent gene expressions have been described in distinct latency stages, which mainly containing four patterns of EBV latent infections [5,6].

EBV infection of Nasopharyngeal carcinoma (NPC) in the type II latency program, where tumor cells typically express the EBV-encoded proteins of EBNA1, LMP1 and LMP2, all of which play a main role in preserving EBV infection and prompting the rest B lymphocytes persistent hyperplasia [7,8,9,10,11]. EBNA1 is a dominant target for CD4^+^ T cells, which consist of 641 amino acids. The protein is a phosphorylated DNA-binding protein that contains multiple glycine and alanine repeats (Gly-Gly-Ala), which may block the treatment and presentation of human leukocyte antigen (HLA) class I-restricted T cells [12,13,14]. LMP1 is a transmembrane protein consisting of 386 amino acids. It is an oncogenic product of EB virus latent membrane protein gene (BNLF1) in the viral genome, which has heterogeneity among the virus strains [15,16]. LMP1 is poorly immunogenic, while the latent membrane protein 2 (LMP2) proteins are immunogenic and thus are ideal targets for EBV-associated immunotherapy [17,18,19]. The Epstein-Barr virus latent membrane protein 2 (*EBV LMP2*) gene encodes the latent membrane protein without carcinogenic effect on tumor cells. Moreover, its antigenic determinant is consistent in different virus strains, existing in tumor pathological specimens and the contained peptides specifically recognizing cytotoxic T lymphocytes (CTL), and inducing a significant specific cellular immune response [8]. Redchenko demonstrated that LMP2 induced a strong specific CTL immune response with dendritic cells (DCs) carrying LMP2 polypeptides, suggesting that LMP2 was an effective target antigen for EBV-associated tumor therapy [20,21]. Hence, LMP2 is a putative target for EBV-related malignancies immunotherapy.

The *LMP2* gene encodes two isoform of membrane proteins, namely LMP2A and LMP2B [22]. Both proteins contain a cytoplasmic C-terminus, which have a similar 12 transmembrane domains and 27 amino acids. LMP2A is a phosphorylated membrane protein with 8 tyrosine at its amino terminus. It interacts with the cellular protein tyrosine kinases Lyn, Syk with SH2-phosphotyrosine, and five proline-rich regions [23,24]. In addition, LMP2A also regulates the proliferation and differentiation of lymphocytes through the ubiquitous Writ and Notch pathways, maintains the EBV virus in latency infections and effectively expresses in the most restricted latent patterns, which suggesting that LMP2 play an important role in EBV latency infections [25]. EBV-LMP2A is considered as an important antigen of EBV-related malignancies and recognized by CTL for multiple epitopes spanning the entire membrane [26,27]. Meanwhile, many vector vaccines targeting LMP2A have been constructed in recent years. However, the function of LMP2B remains an enigma given the lack of appropriate detection methods. Recently, it is speculated that LMP2B may be related to the regulation of LMP2A activity [28,29]. Therefore, our study used the LMP2A protein to construct tumor model cell aimed to evaluate the specific anti-tumor effect of the LMP2-target vaccine. 

Gaussia luciferase (GLuc) is the smallest naturally secreted luciferase, and its fluorescence intensity is 100 times higher than renilla luciferase, making it easier to visualize in live animals [30]. Compared with firefly luciferase, its molecular weight is smaller, and thus its cDNA is easier to insert into expression vectors, and it has a longer half-life. *GLuc* can be observed in living cells and animals in real time with significant signal intensity [31]. The internal ribosome entry site (*IRES)* sequence inserted between the *LMP2* and *GLuc* genes was designed to ensure the independent and stable expression of these exogenous genes in our model cells.

In this study, TC-1-GLUC-LMP2 model tumor cells were constructed to stably and efficiently express LMP2 and *GLuc* to provide candidate model tumor cells to evaluate EBV LMP2-associated tumor vaccines.

## 2. Materials and Methods

### 2.1. Cells and Mice

TC-1 cells used were C57BL/6 mouse lung epithelial cells (American tissue culture collection (ATCC) accession number: CRL-2493), 293 cells were human renal epithelial cells (HEK-293; ATCC accession number: CRL-1573), and 293T cells were human embryonic kidney cells (ATCC accession number: CRL-3216). All cells were maintained in dulbecco’s modified eagle medium (DMEM; HyClone, Logan, UT, USA) containing fetal bovine serum (Gibco, Carlsbad, CA, USA) and penicillin/streptomycin (HyClone) and were then incubated at 37 °C in 5% CO_2_. All cells were provided by our laboratory.

Four to six weeks old female specific pathogen free (SPF)-free C57BL/6 mice were purchased from the Military Academy of Medical Sciences Animal Center (Beijing, China) and maintained under pathogen-free conditions at the animal facilities of the National Institute for Viral Disease Control and Prevention. Mice were sacrificed by cervical dislocation. All animal-related experiments in this study were approved by the Animal Experimental Ethics Committee of National Institute for Viral Disease Control and Prevention, Chinese Center for Disease Control and Prevention (No. 20161122029; the permission date is 16 November 2016).

### 2.2. TC-1-GLUC-LMP2 Cell Line Construction

To amplify *LMP2A* (GenBank accession number: AM746938.1), *IRES*, and *GLuc* (GenBank accession number: LC150601.1) full-length cDNA sequences were obtained from the plasmid of pVR-LMP2 (provided by our laboratory), pLVX-IRES-Puro (Clontech, Mountain View, CA, USA, #632183), and pCMV-Gaussia LUC (Thermo Fisher Scientific, Waltham, MA, USA, #16147) [32], respectively. The following primers were used: Forward LMP2F: 5′-CCGGAATTCCGGATGGGGTCCCTAGAAATGGTG-3′; Reverse LMP2R: 5′-GGAGGGAGAGGGGCTTATACAGTGTTGCGATATGGGG-3′. Forward IRESF: 5′-CATATCGCAACACTGTATAAGCCCCTCTCCCTCCC-3′; Reverse IRESR: 5′-CAACAGAACTTTGACTCCCATTTATCATCGTGTTTTTCAAAGGAAAACC-3′. Forward GLucF: 5′-GGTTTTCCTTTGAAAAACACGATGATAAATGGGAGTCAAAGTTCTGTTTG-3′; and Reverse GLucR: 5′-GCTCTAGAGCTTAGTCACCACCGGCCC-3′.

The recombinant plasmid pLVX-GLUC-LMP2 was constructed using the Lenti-X HTX lentiviral packaging system (Clontech, Mountain View, CA, USA) and was transfected into 293T cells for 72 h to establish the LV-GLUC-LMP2 recombinant lentivirus, which was then used to infect the TC-1 cells. TC-1-GLUC-LMP2 clones were selected with 8 μg/mL puromycin (Invitrogen, Carlsbad, CA, USA, #A1113803).

### 2.3. PCR and Reverse-Transcription PCR (RT-PCR)

Genomic DNA from TC-1-GLUC-LMP2 and TC-1 cells were extracted with a Genomic DNA Mini Kit (QIAGEN, Hilden, Germany, #51306), and EBV LMP2 and GLuc primers were designed to detect the *LMP2* and *GLuc* genes in TC-1-GLUC-LMP2 cells: LMP2F: 5′-TGGGGTCCCTAGAAATGG-3′; LMP2R: 5′-CTTATACAGTGTTGCGATATGG-3′; GLucF: 5′-ATGGGAGTCAAAGTTCTGTTTGC-3′; and GLucR: 5′-TTAGTCACCACCGGCCC-3′. Polymerase chain reaction amplifications were conducted using (Takara, Toyobo, Tokyo, Japan, #R045A) with 35 cycles of 98 °C for 10 s, 51 °C for 10 s, and 72 °C for 10 s. PCR products were separated on a 1% agarose gel. Moreover, the inserted gene sequences of TC-1-GLUC-LMP2 cell were validated by sequencing (Supplementary Materials).

RNA from TC-1-GLUC-LMP2, TC-1, and 293 cells transfected with pVR-LMP2 and 293 cells transfected with pCMV-Gaussia LUC were extracted using an RNeasy Mini Kit (QIAGEN, #74104). EBV LMP2 and *GLuc* primers were designed to detect *LMP2* and *GLuc* mRNA in TC-1-GLUC-LMP2 cells: LMP2F: 5′-GATGGCGGAAACAACTCCC-3′; LMP2R: 5′-GGAACAGTCGTGCCAGAAG-3′; GLucF: 5′-ATCGTGGCCGTGGC-3′; and GLucR: 5′-GCCCTTTGAGGCAGCC-3′. Amplification was performed using a TaKaRa One Step RNA PCR Kit (Takara, Toyobo, Tokyo, Japan, #RR024A) with the reaction at 50 °C for 30 min, the denaturation at 94 °C for 2 min, and 30 cycles at 94 °C for 30 s, 52 °C for 30 s, and 72 °C for 7 min. PCR products were validated with a 1% agarose gel.

### 2.4. Confirming the Stability of Protein

The 1st, 10th and 30th generations of the TC-1-GLUC-LMP2 cells and their supernatants were collected to detect LMP2 and *GLuc* expression. The different generations of TC-1-GLUC-LMP2 and TC-1 cells were separated using a membrane protein extraction kit (KEYGEN BioTECH, Beijing, China, #KGP350), and assayed by Western blot. Membranes were incubated with EBV LMP2 monoclonal antibody (Santa Cruz Biotechnology, Dallas, TX, USA, #sc-101314) diluted 1:500 in 5% skimmed milk, and a horseradish peroxidase-conjugated goat anti-rat IgG (ZSGB-BIO, Beijing, China, #ZB-2307) diluted 1:1000 was used as the secondary antibody. Visualized with a DAB kit (ZSGB-BIO, Beijing, China, #ZLI-9017). The supernatants from different generations of TC-1-GLUC-LMP2 cells were assayed with the BioLux Gaussia Luciferase Assay Kit (Biolink Biotechnology, Beijing, China, #E3300L) and the GLuc protein activity in different generations of TC-1-GLUC-LMP2 cells was tested using the Promega Glomax 96 (Promega, Madison, WI, USA) microplate luminescence detector.

### 2.5. The Purity Detection of TC-1-GLUC-LMP2 Cell Line

The 5 × 10^5^ 30th generation of TC-1-GLUC-LMP2 and TC-1 cells were harvested by centrifugation at 1500 rpm for 5 min, resuspended with by phosphate buffer saline (PBS) containing 1% fatal bovine serum (FBS). Cells were then incubated with the EBV LMP2 monoclonal antibody (Santa Cruz Biotechnology, #sc-101314) diluted 1:100 for 30 min at 37 °C, washed by PBS containing 1% FBS, followed by incubation with 1:100 PBS-diluted fluorescein isothiocyanate (FITC)-labeled rat IgG secondary antibody (ZSGB-BIO, #ZB-0315) for 30 min at 37 °C. The cells were permeabilized using a Cytofix/Cytoperm Solution kit (eBioscience, San Diego, CA, USA, #554714) and analyzed with all six color channels of the BD Biosciences FACSCalibur flow cytometer instrument (BD FACS Calibur, Franklin lake, NJ, USA).

### 2.6. TC-1-GLUC-LMP2 Growth Curve

Cell viability was determined by inoculating 5 × 10^3^ 30th generation of TC-1-GLUC-LMP2 or TC-1 cells on ACEA Biosciences E-Plate 8 plates (ACEA Biosciences Inc., San Diego, CA, USA). Real time cellular analysis (RTCA) iCELLigence (ACEA Biosciences Inc.) was then used to dynamically test the cell index (CI), collect monitoring data and plot cell growth curves.

### 2.7. In Vitro Killing of LMP2-Targeted Model Cells

TC-1-GLUC-LMP2 cells were seeded at 5 × 10^3^ cells/well in E-Plate 8 plates (ACEA Biosciences Inc.) and cultured for 1 h. Splenic lymphocytes were isolated from C57BL/6 mice immunized with the vaccine-LMP2 (vaccine with the *LMP2* gene, MVA-LMP2, provided by our lab; 10^7^ PFU per mouse) and vaccine-NULL (vaccine without the *LMP2* gene, MVA-NULL). Next, splenocytes were spiked with 10 μg/mL IL-2 (Peprotech, London, UK, #212-12) and LMP2-specific peptide, subjected to doubling dilutions from 5 × 10^4^ to 8 × 10^5^ and respectively mixed with target cells that were seeded on the E-Plate 8 plates to detect the cell index (CI) of TC-1-GLUC-LMP2 with the iCELLigence analyzer (ACEA Biosciences Inc.). In addition, 8 × 10^5^ spleens isolated from mice immunized with the vaccine-LMP2 without LMP2 peptides stimulation mixed with TC-1-GLUC-LMP2 cells were set as the non-peptide control.

### 2.8. In Vivo Detection of Tumor Cells

Seven four weeks old female C57BL/6 mice were subcutaneously inoculated with 4 × 10^6^ TC-1-GLUC-LMP2 cells per mouse. Chloral hydrate (Solarboi Life Sciences, Beijing, China) was used as an anesthesia before injecting the substrate coelenterazine H (YEASEN, Shanghai, China, #40906ES02). The in vivo imaging system (IVIS; Xenogen, Alameda, CA, USA) was used to detect the tumor sizes after TC-1-GLUC-LMP2 cells had been inoculated for three, seven, and 14 days. The number of photons and the weights of the tumors were recorded, and the volume was calculated by the formula: (width^2^ × 0.5 × length) for 14 days [33].

### 2.9. Immunohistochemistry and Hematoxylin-Eosin (HE) Staining

After the injected tumor cells had grown for 14 days, tumor tissues were isolated and fixed in paraformaldehyde at 4 °C. Paraffin-embedded sections were stained with hematoxylin-eosin (ZSGB-BIO). The EBV LMP2 monoclonal antibody (Santa Cruz Biotechnology) diluted 1:50 in PBS was added after the sections were washed in an ethanol gradient. A horseradish peroxidase-labeled goat anti-rat IgG antibody (ZSGB-BIO) diluted 1:50 in PBS was used as the secondary antibody, and staining was performed with a solution containing DAB (3,3′-diaminobenzidine).

### 2.10. TC-1-GLUC-LMP2 Tumor Challenge

Ten C57BL/6 mice were randomly divided into two groups. Five mice from one group were intramuscularly immunized with 2 × 10^7^ PFU of vaccine-LMP2 (provided by our lab) and another group injected 2 × 10^7^ PFU of vaccine-NULL (provided by our lab), followed by one booster immunization two weeks later. Ten days after the last immunization, the mice were subcutaneously inoculated with 4 × 10^6^ TC-1-GLUC-LMP2 tumor cells. The size of the tumor cells in mice was observed at 3, 7 and 14 days after inoculation with the IVIS imaging system (Xenogen, Alameda, CA, USA).

Fourteen days after TC-1-GLUC-LMP2 inoculation, the spleen lymphocytes of C57BL/6 mice were collected and the effect of the EBV LMP2 specific immune response detected by enzyme-linked immunospot assay (ELISPOT). Mouse splenic lymphocytes (1 × 10^6^ cells/well) were seeded into a 96-well polyvinylidene fluoride (PVDF)-membrane plate (Merck, Darmstadt, Germany) coated with mouse IFN-γ antibody (Mabtech, Stockholm, Sweden) and 10 μg/mL LMP2-specific peptide, 1:1000 diluted anti-IFN-γ secondary antibody (Mabtech) with PBS containing 1% FBS (50 μL) was added to each well. After 2 h, 1:1000 diluted streptavidin-ALP with PBS containing 1% FBS (Mabtech) was added to each well, followed by a 100 μL of 2-amino-2-methyl-1-propanol (BCIP/NBT-plus; Mabtech) substrate per well. Enzyme-linked spot analysis (AID, Bielefeld, Germany) detected dot-derived cells (SFCs).

### 2.11. Statistical Analysis

All statistical analyses were performed with GraphPad Prism 7.0 software (GraphPad Software Inc., La Jolla, CA, USA). Statistical data were analyzed using unpaired *t*-test with Welch’s correction for unequal variance.

## 3. Results

### 3.1. TC-1-GLUC-LMP2 Construction

**Figure 1:**
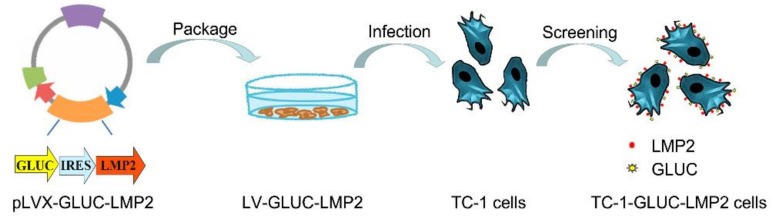
Schematic demonstrating TC-1-GLUC-LMP2 cell construction. Latent membrane protein 2 (*LMP2*) genes, internal ribosome entry site (*IRES*) and *Gaussia luciferase* (*GLuc*) sequences were inserted into TC-1 cells.

To detect the tumor cells, we first constructed TC-1-GLUC-LMP2 cells. *LMP2*, *IRES*, and *GLuc* sequences were inserted into the pLVX plasmid to construct the pLVX-GLUC-LMP2 recombinant plasmid. A recombinant lentivirus carrying the *LMP2* and *GLuc* genes was obtained by the Lenti-X HTX lentiviral packaging system and was used to infect TC-1 cells. TC-1-GLUC-LMP2 tumor cells stably expressing *LMP2* and *GLuc* were selected by puromycin. Figure 1 shows the cell construction protocol.

### 3.2. LMP2 and GLuc Genes Validation in TC-1-GLUC-LMP2 Cells

**Figure 2:**
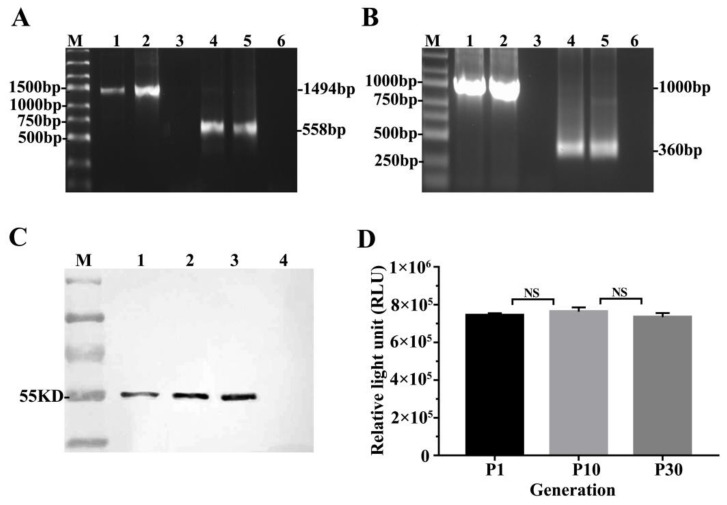
The LMP2 and *GLuc* detection result in TC-1-GLUC-LMP2 cells. (**A**) PCR results of *LMP2* and *GLuc* amplification using the TC-1-GLUC-LMP2 cells genomic DNA as a template. M: DNA ladder; 1: TC-1-GLUC-LMP2 cells as template using primers for *LMP2*; 2: DNA of pVR-LMP2 plasmid; 3: genomic DNA of TC-1 cells; 4: TC-1-GLUC-LMP2 cells as template using primers for GLuc; 5: DNA of pCMV-Gaussia Luc; 6: genomic DNA of TC-1 cells; (**B**) RT-PCR results to verify *GLuc* and *LMP2* mRNA expression. M: RNA ladder; 1: RT-PCR result using TC-1-GLUC-LMP2 as template amplifying *LMP2*; 2: 293 cells transfected with pVR-LMP2 as template amplifying *LMP2*; 3: TC-1 cells as template amplifying *LMP2;* 4: RT-PCR result using TC-1-GLUC-LMP2 as template amplifying *GLuc*; 5: 293 cells transfected with pCMV-Gaussia Luc as template amplifying *GLuc*; 6: TC-1 cells as template amplifying *GLuc*; (**C**) Western blot results of LMP2 protein expression in TC-1-GLUC-LMP2 cells at different passages. M: PageRuler Prestained Protein Ladder; 1: Passage 1 TC-1-GLUC-LMP2 cells, LMP2 with a molecular weight of 55 KD; 2: Passage 10 TC-1-GLUC-LMP2 cells; 3: Passage 30 TC-1-GLUC-LMP2 cells; 4: TC-1 cells; (**D**) Identifying the stable expression of *GLuc*. The mean relative light unit (RLU) value of 10^4^ TC-1-GLUC-LMP2 cells at passages 1, 10 and 30 were 7.44 × 10^5^, 7.63 × 10^5^ and 7.34 × 10^5^. NS, no significant difference; each column represents mean ± SD (*n* = 5) in (**D**).

We intend to detect the *LMP2* and *GLuc* genes existed in the TC-1-GLUC-LMP2 cells by PCR, the results showed that the full-length amplified fragments of latent membrane protein 2 (*LMP2*) and *Gaussia luciferase* (*GLuc*) were 1494 bp and 558 bp, respectively, as shown in Figure 2A. The RT-PCR results showed that the amplified fragments from the exogenous genes were 1000 bp and 360 bp, respectively, as shown in Figure 2B. These PCR results showed that the *LMP2* and *GLuc* genes were successfully inserted into the TC-1-GLUC-LMP2 genome. The inserted sequences resulting from the TC-1-GLUC-LMP2 cell was validated by sequencing (Supplementary Materials).

Next, we wanted to determine whether the LMP2 and *GLUC* proteins could stably express in TC-1-GLUC-LMP2 cells. Western blot was used to detect the LMP2 expression of the exogenous genes in TC-1-GLUC-LMP2 cells at different passages. The results showed that the first generation of TC-1-GLUC-LMP2 cells expressed LMP2 protein with a molecular weight of 55 KD, consistent with the results from the 10th and 30th generation TC-1-GLUC-LMP2 cells. The TC-1 cells did not express LMP2 protein (Figure 2C). The relative light unit (RLU) results for *GLuc* activity in the 1st, 10th and 30th generations of the TC-1-GLUC-LMP2 cells were 7.44 × 10^5^, 7.63 × 10^5^ and 7.34 × 10^5^, respectively. These results indicated that the *LMP2* and *GLuc* proteins were stably expressed in TC-1-GLUC-LMP2 cells for at least 30 generations (Figure 2D).

### 3.3. The Purity of TC-1-GLUC-LMP2 Cell Line

**Figure 3:**
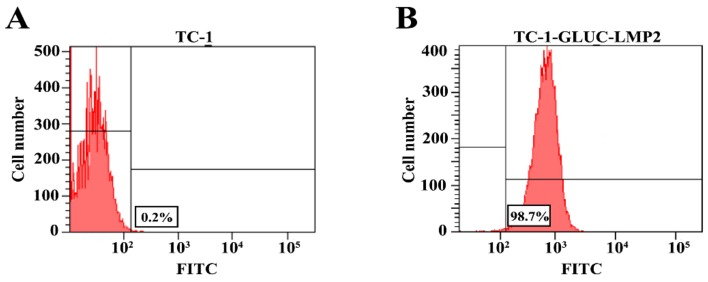
Flow cytometry detection results. (**A**) The proportions of fluorescein isothiocyanate (FITC)-labeled cells in TC-1 was 0.2%; (**B**) The proportions of FITC-labeled cells in TC-1-GLUC-LMP2 was 98.7%. FITC was fluorescein isothiocyanate. The red area represented the detected cells.

To analyze the purity of TC-1-GLUC-LMP2, TC-1 and the 30th generation TC-1-GLUC-LMP2 cells were detected by flow cytometry, and the results showed that the proportion of FTTC-labeled TC-1-GLUC-LMP2 cells was 98.7% (Figure 3B), suggesting that almost all TC-1-GLUC-LMP2 tumor cells stably and efficiently expressed the LMP2 protein.

### 3.4. TC-1-GLUC-LMP2 Growth Curve

**Figure 4:**
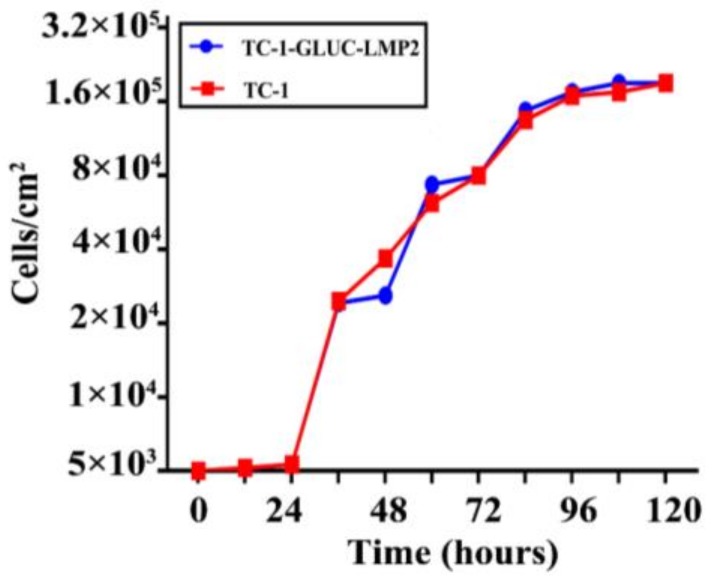
Comparing the TC-1-GLUC-LMP2 and TC-1 growth curves. TC-1-GLUC-LMP2 and TC-1 cells (5 × 10^3^) were added to E-plate L 8 plates, and cell index (CI) was measured by iCELLigence. After 120 h, both cell lines grew to 2.1 × 10^5^/cm^2^. The growth rate and adhesion were not significantly different.

In this study, we further examined the cell activity and proliferation of TC-1-GLUC-LMP2. Figure 4 shows the growth curve of TC-1-GLUC-LMP2 and TC-1 cells, which increased from 5 × 10^3^ to 2.1 × 10^5^ after 120 h, indicating that the *EBV LMP2* and *GLuc* gene insertion did not affect the growth characteristics of TC-1 cells.

### 3.5. Immunogenicity of TC-1-GLUC-LMP2 Cells

**Figure 5:**
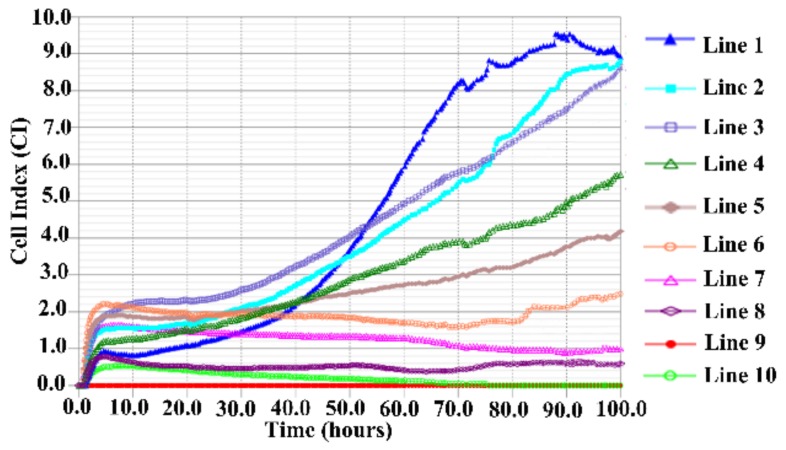
Cytotoxicity result of TC-1-GLUC-LMP2 cells. The ten groups of mixed cells were inoculated on E-plate L8 plates and incubated for 100 h. Line 1: 5 × 10^3^ TC-1-GLUC-LMP2 target cells; Line 2: 5 × 10^3^ TC-1-GLUC-LMP2 target cells mixed with 8 × 10^5^ spleens without peptide stimulation isolated from mice immunized with vaccine-LMP2; Line 3: 5 × 10^3^ target cells mixed with 8 × 10^5^ spleens were spiked with LMP2-specific peptide isolated from mice immunized with vaccine-NULL; Line 4: 5 × 10^3^ TC-1-GLUC-LMP2 target cells mixed with 5 × 10^4^ spleens with peptide stimulation isolated from mice immunized with vaccine-LMP2; Line 5: 5 × 10^3^ target cells mixed with 1 × 10^5^ spleens with peptide stimulation isolated from mice immunized with vaccine-LMP2; Line 6: 5 × 10^3^ target cells mixed with 2 × 10^5^ spleens with peptide stimulation isolated from mice immunized with vaccine-LMP2; Line 7: 5 × 10^3^ target cells mixed with 4 × 10^5^ spleens with peptide stimulation isolated from mice immunized with vaccine-LMP2; Line 8: 5 × 10^3^ target cells mixed with 8 × 10^5^ spleens with peptide stimulation isolated from mice immunized with vaccine-LMP2; Line 9: Roswell Park Memorial Institute 1640 (RPMI 1640); Line 10: 8 × 10^5^ LMP2-specific mouse spleen lymphocytes.

In this study, we investigate whether TC-1-GLUC-LMP2 target cells could be effectively killed by LMP2-specific CTLs in vitro. The iCELLigence analyzer is an automated cell analysis system, providing a convenient and efficient method for acquiring real-time dynamic information. Due to the cell adhesion and proliferation on the electrodes, current flow is blocked, providing very sensitive readings of cell numbers. LMP2-specific mouse spleen lymphocytes (2-fold diluted from 5 × 10^4^ to 8 × 10^5^) were respectively mixed with 5 × 10^3^ TC-1-GLUC-LMP2 target cells. The iCELLigence results showed that when 8 × 10^5^ spleen lymphocytes isolated from mice injected with vaccine-LMP2 (provided by our lab) were mixed with TC-1-GLUC-LMP2 cells, the target cell attachment rate was significantly reduced, indicating that the target cells were effectively killed by LMP2-specific CTLs (Figure 5, Line 8). In addition, the specific killing effect was obviously increased by improved quantities of LMP2-peptide stimulated spleens (Figure 5, Line 4–8). The 8 × 10^5^ spleens isolated from mice immunized with the vaccine without the *LMP2* gene (vaccine-NULL, provided by our lab) were spiked with LMP2-specific peptide and mixed with 5 × 10^3^ TC-1-GLUC-LMP2 cells (Figure 5, Line 3), which had no significant effect on the target cells as there was no significant difference when compared with the TC-1-GLUC-LMP2 cells alone in the cells index (CI; Figure 5, Line 1). Moreover, the cell index of 8 × 10^5^ spleens separated from mice injected vaccine-LMP2 (provided by our lab) without LMP2-specific peptide stimulation mixed with 5 × 10^3^ TC-1-GLUC-LMP2 cells was very similar to that of the TC-1-GLUC-LMP2 cells alone (Figure 5, Line 1, 2). The 8 × 10^5^ LMP2-specific mouse spleen lymphocytes (SPLs; Figure 5, Line 10) and Roswell Park Memorial Institute 1640 (RPMI 1640, Hyclone; Figure 5, Line 9) caused no defects in cell attachment, thus the CI curve was straight. These data indicated that the TC-1-GLUC-LMP2 target cells could be effectively killed by LMP2-specific CTLs, and the specific growth ability with the quantities of LMP2-based spleens gradually increased.

### 3.6. In Vivo Detection of Tumor Formation

**Figure 6:**
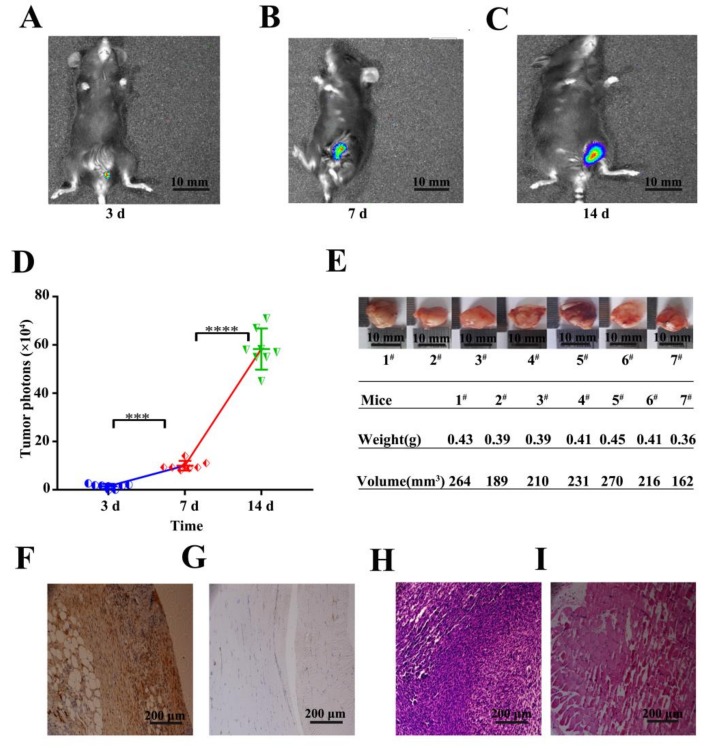
Detecting subcutaneously injected TC-1-GLUC-LMP2 cells. (**A**) The tumor in vivo was observed by the in vivo imaging system (IVIS) at 3 days post-inoculation; (**B**) The tumor was observed by the IVIS at 7 days post-inoculation; (**C**) The tumor was observed by the IVIS at 14 days post-inoculation; (**D**) IVIS results showed that the mean number of photons in the mice were 2.43 × 10^4^, 9.67 × 10^4^ and 7.09 × 10^5^ at three, seven, and 14 days post-inoculation, respectively; (**E**) The tumor sizes and weights of mice were recorded at 14 days after inoculation with the tumor cells; the average tumor weight and volume of the tumor-bearing mice were 0.41 g and 220 mm^3^, respectively; (**F**) Immunohistochemistry results for TC-1-GLUC-LMP2 tumors showed nuclei (blue) and cytoplasm (reddish brown), indicating that EBV LMP2 was well expressed; (**G**) Immunohistochemistry results for normal muscle tissue of mice; (**H**) HE staining results of TC-1-GLUC-LMP2 tumors showed that nuclei became larger (blue), while the cytoplasm shrank or even disappeared (pink); (**I**) HE staining results of normal muscle tissue of mice. # means the number of each mouse. *** *p* < 0.005; **** *p* < 0.001 in (**D**) (*n* = 7).

To detect whether the TC-1-GLUC-LMP2 cells could survive and proliferate in vivo, we inoculated each mouse with 4 × 10^6^ TC-1-GLUC-LMP2 cells, and the in vivo imaging system (IVIS) was used to observe tumor sizes in mice at three, seven and 14 days post-inoculation (Figure 6A–C). As shown in Figure 6D, the IVIS was used to observe tumor sizes in mice at three, seven, and 14 days post-inoculation, and the IVIS results showed that the photon number increased with increased inoculation time, suggesting that the tumor volumes were constantly increasing. After 14 days, the tumors were isolated, observed, and weighed. As shown in Figure 6E, the average tumor weight and volume were 0.41 g and 220 mm^3^, respectively.

Next, we wanted to examine the LMP2 expression in the TC-1-GLUC-LMP2 cells and made sure that the TC-1-GLUC-LMP2 could form tumor tissue in vivo. The immunohistochemistry results of the TC-1-GLUC-LMP2 tumor tissues showed reddish-brown staining, while the normal muscle tissue was colorless, suggesting that the tumor formed by the TC-1-GLUC-LMP2 cells expressed LMP2 protein (Figure 6F,G). HE staining showed that the TC-1-GLUC-LMP2 tumor cell nuclei became larger while the cytoplasm shrank, which is significantly different to normal muscle tissue and typical of tumor cells (Figure 6H,I). These results showed that TC-1-GLUC-LMP2-injected mice had significantly increased tumor volumes over time. Moreover, the TC-1-GLUC-LMP2 cells that formed tumors expressed LMP2 protein in vivo.

### 3.7. Tumor Challenge

**Figure 7:**
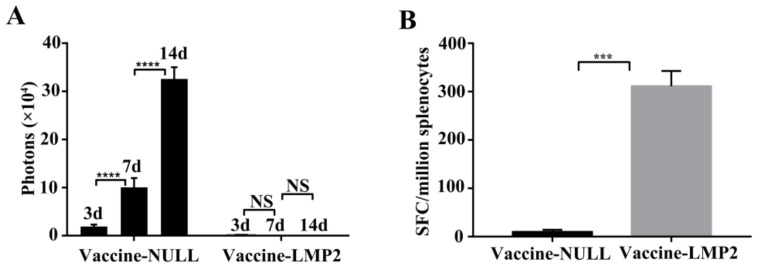
TC-1-GLUC-LMP2 tumor challenge with LMP2-associated vaccine in vivo. (**A**) The number of tumor photons in the vaccine-NULL or vaccine-LMP2-immunized mice were detected at three, seven, 14 days with the in vivo imaging system (IVIS); (**B**) The LMP2-specific immune response was analyzed by ELISPOT after TC-1-GLUC-LMP2 tumor cells inoculation 14 days. *** *p* < 0.005; **** *p* < 0.001; NS, no significant difference; each column represents mean ± SD (*n* = 5).

We want to analyze whether the TC-1-GLUC-LMP2 cells could be effectively suppressed and killed in vivo, ten days after immunized mice with the vaccine-NULL or the vaccine-LMP2, we inoculated TC-1-GLUC-LMP2 tumor cells and observed the photon numbers of tumor cells three, seven, and 14 days after inoculation. Fourteen days after inoculation with tumor cells, the results of ELISPOT showed that vaccine-LMP2 induced significantly LMP2-specific immune responses (Figure 7B). The tumors disappeared at three days post-tumor cell inoculation in five mice with the vaccine-LMP2 immunization (Figure 7A). However, the results of ELISPOT of the mice immunized with vaccine-NULL suggested that there was little induction of LMP2-specific immune responses (Figure 7B). Meanwhile, the number of photons in mice injected with the vaccine-NULL was observed at three days after tumor cell inoculation, and the number of tumor photons significantly increased with the inoculation time (Figure 7A; *p* < 0.001). Together, these results suggest that TC-1-GLUC-LMP2 could be efficiently inhibited and cleared by the LMP2-specific immune responses induced by the LMP2-related vaccine in vivo.

## 4. Discussion

EBV is widespread and infects more than 90% of the worldwide population [25]. Among non-immunocompromised patients, highly pathogenic latent EBV infections correlate with severe malignancies such as gastric cancer, nasopharyngeal cancer, Burkitt’s lymphoma and Hodgkin’s lymphoma [4,34,35] At present, radiation and combination chemotherapy are the basic methods for the treatment of Nasopharyngeal carcinoma (NPC). The five-year survival rates of NPC patients in domestic and foreign literature have been reported as 50–60%. NPC treatment failure is mostly locally-aggressive recurrence and distant metastasis. The distant metastasis rate of NPC post-treatment ranged from about 22% to 36% [7]. With the development of molecular biology and tumor immunology, the application of immunotherapy to prevent recurrence and distant metastasis of NPC as a supplement to traditional treatment methods has gradually gained attention. EBV-related immunotherapy is primarily based on EBV-antigens-targeted therapy in tumors [36]. Cellular immunity plays a significant role in the clearance of virus infection and tumor immune surveillance. The T lymphocytes of NPC have certain specific killing and suppressing effects on NPC cancer cells. It has been reported that the induced EBV-specific immune responses of NPC patients and EBV-IgA-VCA-positive individuals were decreased compared with normal people, suggesting that the EBV-specific cellular immune responses may be associated with the immunotherapy of NPC [37]. Since the immune function of NPC patients in a long-term low status, the application of radiation and chemotherapy may lead to a further decline in immunity, thus the use of targeted immunotherapy is considerably significant to fill the gap of traditional treatment methods. Currently, there are two methods for improving the specific CTL responses. The first method is adoptive CTL treatment, which inputs specifically activated self or allogeneic HLA-matched CTL into the patient [38]. However, this method is time-consuming and expensive, the CTL of allogenic origin still has problems with HLA matching and biological safety, and difficult to promote in a wide range of people. The second method is vaccination, one of which uses the EBV antigen or a definite CTL epitope activating the immunization responses of patients and thereby enhancing the specific immune effects. Steven screened a series of LMP2 HLA class I restricted epitopes through CTL-CTL cytotoxicity experiments [39]. Dendritic cells with efficient presenting ability could be isolated and modified with peptides, and then translocated into the body to stimulate the activation and proliferation of CTL memory cells [40,41]. Unfortunately, this method has the same disadvantages with the adoptive treatment methods. Another strategy is the use of molecular biology techniques, introducing the *LMP2* gene into the NPC patient, expressing the endogenous LMP2 protein and activating its own specific memory CD8^+^ T cells. With the specific immune responses improving, reaching the goal of preventing and treating cancer [42,43]. In recent years, viral vectors have played a gradual role in the field of targeted immunotherapy and recombinant vaccine development such as adenovirus, poxvirus, and retrovirus. The successful establishment of antigens-targeted recombinant vector vaccines such as the adeno-associated virus (AAV)-LMP2, recombinant adenovirus (rAd)-EBV-LMP2, and modified vaccinia virus Ankara (MVA)-EL vaccines, all of which induce significantly specific cellular immune responses, has provided many promising vaccine candidates for the treatment of EBV-related malignancy [32,44,45]. However, at present, the specific immune response evaluation of LMP2 targeted vaccines have mainly been regarding the induction of cellular and humoral immune response effects, while the field of LMP2-specific tumor challenge ability assessment is still vacant. Therefore, our study constructed a tumor cell expressing the LMP2 antigen, effectively evaluated the anti-tumor ability of LMP2-related vaccine, and improved the assessment system of the LMP2-associated vaccine.

As above-mentioned, it is essential to construct target tumor cell models. Currently, there are significant EBV-related tumor cell model-related research and animal model systems to assess the effects of vaccine-targeted treatments. The construction of TC-1 tumor cell lines that express EBV LMP1 has been used to evaluate the anti-tumor and cytotoxic effects of preclinical vaccines [46]. TC-1 tumor cells have been widely used to construct model tumor cells and HPV16-18 model tumor cells with the exogenous genes efficientive expression over many passages. Furthermore, these model tumor cells can be specifically killed by the CD8^+^ T cell-dependent cellular immune response to assess the anti-tumor immune responses of vaccines [47,48]. Moreover, TC-1 model cells can be used not only to evaluate the H9N2 avian influenza virus immune response, but also to provide useful information for the H9N2 virus-host immune interaction [49]. These cells have been used as the tumor cell model to evaluate vaccine-specific immune responses and anti-tumor effects in a wide range of applications, and the feasibility of these experiments is highlighted in this work.

Establishing model tumor cells is particularly significant for evaluating EBV LMP2-associated vaccines. We constructed TC-1-GLUC-LMP2 model tumor cells that exogenously expressed LMP2 and *GLuc* protein. The western blot results showed that the cells efficiently expressed the EBV LMP2 protein for at least 30 generations, and there was no difference between the passaged cells and the primary tumor cells, suggesting that the constructed model tumor cells stably expressed the transgenes. Increasing the number of passages had no influence on the expression of exogenous gene products. The TC-1-GLUC-LMP2 growth curves showed that the TC-1-GLUC-LMP2 and TC-1 cells had the same growth rate and adhesion, suggesting that the insertion of exogenous *LMP2* and *GLuc* did not affect the growth characteristics of tumor cells. The in vivo imaging system (IVIS) results of tumor-bearing mice immunized with TC-1-GLUC-LMP2 showed that the number of photons gradually increased over 14 days. This indicated that the tumor cells gradually proliferated over time after the tumor cells were inoculated in mice. Finally, the TC-1-GLUC-LMP2 tumor cells were mixed with LMP2-specific mouse splenic lymphocytes. The cell index (CI) of the TC-1-GLUC-LMP2 target cells mixed with LMP2-specific mouse splenic lymphocytes showed an obvious decline (Figure 5, Line 8), and the CI of the TC-1-GLUC-LMP2 target cells (Figure 5, Line 1) was significantly higher than the CI after killing. This suggested that the specific immune response induced by the LMP2-associated vaccine could effectively kill TC-1-GLUC-LMP2 model tumor cells. As the incubation time increased with the LMP2-specific effector cells, the number of target cells decreased, indicating significantly improved cytotoxicity for the target cells. We further concluded that increasing the number of effector cells could reduce the time required to clear the TC-1-GLUC-LMP2 cells and strongly improve the killing effects (Figure 5, Lines 4–8). Furthermore, the LMP2-specific immune responses in the mice immunized with the vaccine-LMP2 were mainly induced by the CD8^+^ T cell (Supplementary Materials Figure S1). In addition, the mice inoculated with TC-1-GLUC-LMP2 tumor cells could be effectively and specifically killed by the immune responses induced by the LMP2-associated vaccine (Figure 7). These data above indicate that the TC-1-GLUC-LMP2 tumor cells could be significantly eliminated by the specific immune response induced by the LMP2-associated vaccine in mice, which further confirm that the feasibility of TC-1-GLUC-LMP2 as target cells to assess the antitumor ability of the EBV LMP2-related vaccine.

In conclusion, we successfully constructed TC-1-GLUC-LMP2 model tumor cells that carried *EBV LMP2*, and *GLuc* exogenously. These cells are an effective model cell to evaluate EBV LMP2-related vaccine-mediated tumor cell killing. Thus, this model provides a useful target cell for the nasopharyngeal carcinoma vaccine. Moreover, it is possible to observe the killing of tumor cells in vitro and in vivo, which can be a reference for the preclinical anti-tumor efficacy evaluation of EBV LMP2-dependent vaccines.

## Supplementary Materials

Supplementary materials can be found at http://www.mdpi.com/1999-4915/10/4/145/s1.
