# Supplementary material for: Constructing TC-1-GLUC-LMP2 Model Tumor Cells to Evaluate the Anti-Tumor Effects of LMP2-Related Vaccines
Source: Viruses. 2018 Mar 23;10(4):145. doi: 10.3390/v10040145 (PMC5923439; doi:10.3390/v10040145)
Supplement: Supplementary file 1 [file viruses-10-00145-s001.pdf]

Supplement for

Constructing TC-1-GLUC-LMP2 Model Tumor Cells to Evaluate the Anti-Tumor Effects of LMP2-Related Vaccines

Liying Sun, Yanzhe Hao, Zhan Wang * and Yi Zeng *

National Institute for Viral Disease Control and Prevention, Chinese Center for Disease Control and Prevention, State Key Laboratory for Infectious Disease Prevention and Control, Beijing 100052, China; sunliying@emails.bjut.edu.cn (L.S.); haoyanzhe@ivdc.chinacdc.cn (Y.H.)

* Correspondence: wangzhan@ivdc.chinacdc.cn (Z.W.); zengycdc@163.com (Y.Z.);
Tel.: +86-010-8354-6064 (Z.W.); +86-010-6355-2662 (Y.Z.);
Fax: +86-010-6358-1345 (Z.W.); +86-010-6355-2661 (Y.Z.)

Received: date; Accepted: date; Published: date

File Supplement 1

Genomic DNA from TC-1-GLUC-LMP2 cells was extracted with a Genomic DNA Mini Kit (QIAGEN, Hilden, Germany, #51306). Primers of the sequences inserted in TC-1-GLUC-LMP2 cells were designed to validate the *LMP2* and *GLuc* genes in TC-1-GLUC-LMP2 cells: F: 5′-AGCGGTTTGACTCACGG-3′; R: 5′-AGTGAGACGTGCTACTTCCA-3′ (Tsingke, Beijing, China). The sequencing result is consistent with the aim gene sequences of 3192 bp, as follows:

5′-AGCGGTTTGACTCACGGGGATTTCCAAGTCTCCACCCCATTGACGTCAATGGG
AGTTTGTTTTGGCACCAAAATCAACGGGACTTTCCAAAATGTCGTAACAACTCCGCCC
CATTGACGCAAATGGGCGGTAGGCGTGTACGGTGGGAGGTCTATATAAGCAGAGCTC
GTTTAGTGAACCGTCAGATCGCCTGGAGACGCCATCCACGCTGTTTTGACCTCCATAG
AAGACACCGACTCTAGCTAGAGGATCGCTAGCGCTACCGGACTCAGATCTCGAGCTC
AAGCTTCGAATTCCGGATGGGGTCCCTAGAAATGGTGCCAATGGGCGCGGTCCCCC
TAGCCCCGGCGGGGATCCGGATGGGTACGATGGCGGAAACAACTCCCAATATCCATC
TGCTTCTGGCTCTTCTGGGAACACCCCCACCCCACCGAACGATGAGGAACGTGAATC
TAATGAAGAGCCCCCACCGCCTTATGAGGACCCATATTGGGGCAATGGCGACCGTCA
CTCGGACTATCAACCACTAGGAACCCAAGATCAAAGTCTGTACTTGGGATTGCAACA
CGACGGGAATGACGGGCTCCCTCCCCCTCCCTACTCTCCACGGGATGACTCATCTCAA
CACATATACGAAGAAGCGGGCAGAGGAAGTATGAATCCAGTATGCCTGCCTGTAATT
GTTGCGCCCTACCTCTTTTGGCTGGCGGCTATTGCCGCCTCGTGTTTCACGGCCTCAGT
TAGTACCGTTGTGACCGCCACCGGCTTGGCCCTCTCACTTCTACTCTTGGCAGCAGTG
GCCAGCTCATATGCCGCTGCACAAAGGAAACTGCTGACACCGGTGACAGTGCTTACT
GCGGTTGTCACTTTCTTTGCAATTTGCCTAACATGGAGGATTGAGGACCCACCTTTTAA
TTCTCTTCTGTTTGCATTGCTGGCCGCAGCTGGCGGACTACAAGGCATTTACGTTCTGG
TGATGCTTGTGCTCCTGATACTAGCGTACAGAAGGAGATGGCGCCGTTTGACTGTTTG
TGGCGGCATCATGTTTTTGGCATGTGTACTTGTCCTCATCGTCGACGCTGTTTTGCAGC
TGAGTCCCCTCCTTGGAGCTGTAACTGTGGTTTCCATGACGCTGCTGCTACTGGCTTTC
GTCCTCTGGCTCTCTTCGCCAGGGGGCCTAGGTACTCTTGGTGCAGCCCTTTTAACATT
GGCAGCAGCTCTGGCACTGCTAGCGTCACTGATTTTGGGCACACTTAACTTGACTACA
ATGTTCCTTCTCATGCTCCTATGGACACTTGTGGTTCTCCTGATTTGCTCTTCGTGCTCT
TCATGTCCACTGAGCAAGATCCTTCTGGCACGACTGTTCCTATATGCTCTCGCACTCTT
GTTGCTAGCCTCCGCGCTAATCGCTGGTGGCAGTATTTTGCAAACAAACTTCAAGAGT
TTAAGCAGCACTGAATTTATACCCAATTTGTTCTGCATGTTATTACTGATTGTCGCTGG
CATACTCTTCATTCTTGCTATCCTGACCGAATGGGGCAGTGGAAATAGAACATACGGT
CCAGTTTTTATGTGCCTCGGTGGCCTGCTCACCATGGTAGCCGGCGCTGTGTGGCTGA

CGGTGATGTCTAACACGCTTTTGTCTGCCTGGATTCTTACAGCAGGATTCCTGATTTTC
CTCATTGGCTTTGCCCTCTTTGGGGTCATTAGATGCTGCCGCTACTGCTGCTACTACTG
CCTTACACTGGAAAGTGAGGAGCGCCCACCGACCCCATATCGCAACACTGTATAAGC
CCCTCTCCCTCCCCCCCCCCTAACGTTACTGGCCGAAGCCGCTTGGAATAAGGCCGGT
GTGCGTTTGTCTATATGTTATTTTCCACCATATTGCCGTCTTTTGGCAATGTGAGGGCC
CGGAAACCTGGCCCTGTCTTCTTGACGAGCATTCCTAGGGGTCTTTCCCCTCTCGCCA
AAGGAATGCAAGGTCTGTTGAATGTCGTGAAGGAAGCAGTTCCTCTGGAAGCTTCTT
GAAGACAAACAACGTCTGTAGCGACCCTTTGCAGGCAGCGGAACCCCCCACCTGGC
GACAGGTGCCTCTGCGGCCAAAAGCCACGTGTATAAGATACACCTGCAAAGGCGGC
ACAACCCCAGTGCCACGTTGTGAGTTGGATAGTTGTGGAAAGAGTCAAATGGCTCTC
CTCAAGCGTATTCAACAAGGGGCTGAAGGATGCCCAGAAGGTACCCCATTGTATGGG
ATCTGATCTGGGGCCTCGGTGCACATGCTTTACATGTGTTTAGTCGAGGTTAAAAAA
CGTCTAGGCCCCCCGAACCACGGGGACGTGGTTTTCCTTTGAAAAACACGATGATAA
ATGGGAGTCAAAGTTCTGTTTGCCCTGATCTGCATCGCTGTGGCCGAGGCCAAGCCCA
CCGAGAACAACGAAGACTTCAACATCGTGGCCGTGGCCAGCAACTTCGCGACCACG
GATCTCGATGCTGACCGCGGGAAGTTGCCCGGCAAGAAGCTGCCGCTGGAGGTGCTC
AAAGAGTTGGAAGCCAATGCCCGGAAAGCTGGCTGCACCAGGGGCTGTCTGATCTGC
CTGTCCCACATCAAGTGCACGCCCAAGATGAAGAAGTTCATCCCAGGACGCTGCCAC
ACCTACGAAGGCGACAAAGAGTCCGCACAGGGCGGCATAGGCGAGGCGATCGTCGA
CATTCCTGAGATTCCTGGGTTCAAGGACTTGGAGCCCTTGGAGCAGTTCATCGCACAG
GTCGATCTGTGTGTGGACTGCACAACTGGCTGCCTCAAAGGGCTTGCCAACGTGCAGT
GTTCTGACCTGCTCAAGAAGTGGCTGCCGCAACGCTGTGCGACCTTTGCCAGCAAGA
TCCAGGGCCAGGTGGACAAGATCAAGGGGGCCGGTGGTGACTAAGCTCTAGATAATT
CTACCGGGTAGGGGAGGCGCTTTTCCCAAGGCAGTCTGGAGCATGCGCTTTAGCAGC
CCCGCTGGGCACTTGGCGCTACACAAGTGGCCTCTGGCCTCGCACACATTCCACATCC
ACCGGTAGGCGCCAACCGGCTCCGTTCTTTGGTGGCCCCTTCGCGCCACCTTCTACTC
CTCCCCTAGTCAGGAAGTTCCCCCCCGCCCCGCAGCTCGCGTCGTGCAGGACGTGAC
AAATGGAAGTAGCACGTCTCACT-3′.

Supplement 2

Methods

IFN-γ Detection

The splenic lymphocytes isolated from vaccine-LMP2 and vaccine-NULL immunized mice, respectively, were stimulated with anti-mouse CD28 (eBioscience, San Diego, CA, USA), EBV LMP2-specific peptide, and IL-2 at a final concentration of 10 μg/mL (Peprotech, Rocky Hill, NJ, USA). The protease transport inhibitor brefeldin A (Sigma, Japan) and monensin sodium (Amresco, Solon, OH, USA) were added, and samples were incubated for 24 h. In the meantime, lymphocyte negative controls without LMP2-peptide stimulation were set. The splenic lymphocytes were washed with 2% fetal bovine serum (FBS) in PBS and stained for surface markers with various fluorescently tagged monoclonal antibodies: 20 μL each of V450-labeled anti-mouse CD3, PerCP-Cy™5.5-labeled anti-mouse CD4, PE-labeled anti-mouse CD8a, and APC-labeled CD49b. The cells were permeabilized using a Cytofix/Cytoperm Solution kit (eBioscience) and stained with fluorescein isothiocyanate-conjugated anti-mouse IFN-γ (All antibodies were purchased from eBioscience). Samples were analyzed with all six color channels of the BD Biosciences FACSCalibur flow cytometer instrument (BD FACS Calibur, USA).

Result

EBV-LMP2 Specific IFN-γ Analysis

In this study, we wanted to detect the quantities of IFN-γ in CD4$^+$ T, CD8$^+$ T induced by vaccine-LMP2. C57BL/6 mice were immunized with vaccine-LMP2 and vaccine-NULL, respectively, followed by one booster injection 2 weeks later. Ten days after the last immunization, mice splenocytes were harvested for flow cytometry detection. CD3$^+$ T cells were screened by flow cytometry (Figure S1A). The percentages of IFN-γ in the CD3$^+$CD4$^+$ T cells and CD3$^+$CD8$^+$ T cells were detected by flow cytometry, and the results showed that the quantities of IFN-γ in CD3$^+$CD8$^+$ T cells isolated from vaccine-LMP2 immunized mice were significantly higher than those in CD3$^+$CD4$^+$ T cells (Figures S1B and S1C). Moreover, the quantities of IFN-γ in the CD3$^+$CD4$^+$ T cells isolated from vaccine-NULL immunized mice had no significant difference when compared with those separated from vaccine-LMP2 immunized mice (Figures S1C). These data suggested that the vaccine-LMP2 induction of specific immune responses was mainly CD8$^+$ T cell dependent (Figure S1D).

In the meanwhile, we investigated the percentage of IFN-γ in NK cells induced by vaccine-LMP2 or vaccine-NULL. Mice splenocytes were harvested from vaccine-LMP2 and vaccine-NULL immunized mice, respectively, for flow cytometry detection as there was a negative control without LMP2-peptide stimulation. The quantities of IFN-γ in NK cells were detected by the surface marker CD49b of NK cells. The results showed that the release of IFN-γ in NK cells isolated from mice immunized with vaccine-LMP2 mice was scarce as there was no difference with those stimulated without LMP2-peptides and those separated from vaccine-NULL (Figures S1E and S1F). In addition, the percentages of IFN-γ in CD8$^+$ T cells were significantly higher than those in NK cells (Figure S1G). The above data further confirmed that the LMP2-specific immune responses was mainly induced by the CD8$^+$ T cell.

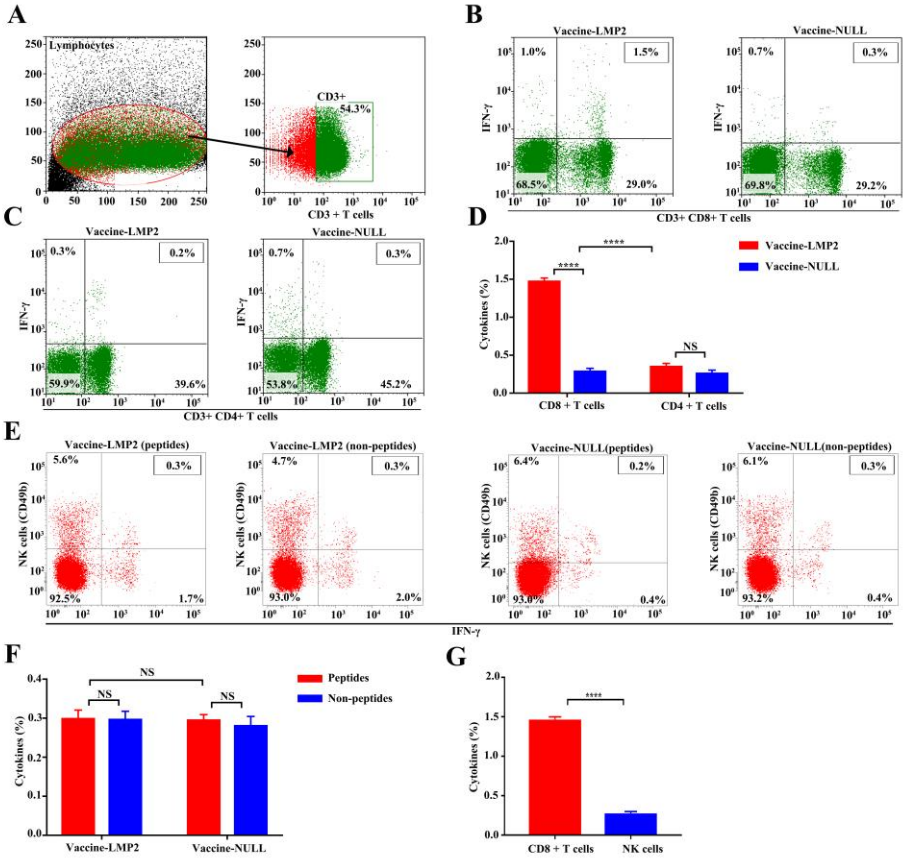

Figure S1. Flow cytometry detection IFN-γ results. (**A**) Flow cytometry gating of $CD3^+$ T cells. (**B**,**C**) The proportions of IFN-γ in $CD3^+CD8^+$ T (**B**) and $CD3^+CD4^+$ (**C**) cells. (**D**) The cytokine percentages of IFN-γ in $CD8^+$ and $CD4^+$ T cells. (**E**) The quantities of IFN-γ in NK cells stimulated with LMP2-peptides isolated from vaccine-LMP2 and vaccine-NULL mice, and those stimulated without LMP2-peptides. (**F**) The cytokine percentages of IFN-γ in NK cells. (**G**) The cytokine percentages of IFN-γ in $CD8^+$ T and NK cells. Each column represents mean ± SD ($n = 5$) in (**D**,**F**,**G**), (**** $p < 0.001$; NS, no significant difference).
